# Methylphenidate enhances or impairs the cognitive control of Pavlovian bias depending on working memory capacity

**DOI:** 10.7554/eLife.98917

**Published:** 2026-06-16

**Authors:** Dirk EM Geurts, Hanneke EM den Ouden, Jennifer C Swart, Monja I Froböse, Sean Fallon, Jennifer L Cook, Roshan Cools

**Affiliations:** 1 Donders Institute for Brain, Cognition and Behaviour, Centre for Cognitive Neuroimaging, Radboud University Nijmegen Netherlands; 2 Department of Psychiatry, Radboud University Medical Center Nijmegen Netherlands; 3 Biological Psychology of Decision Making, Institute of Experimental Psychology, Heinrich Heine University Düsseldorf Düsseldorf Germany; 4 School of Psychology, University of Plymouth Plymouth United Kingdom; 5 School of Psychology and Centre for Human Brain Health, University of Birmingham Birmingham United Kingdom; King's College London United Kingdom; University College London United Kingdom

**Keywords:** Pavlovian-to-instrumental transfer, Pavlovian bias, methylphenidate, catecholamine, inhibition, approach avoidance, Human

## Abstract

Value-based decision making is regulated by a delicate interplay of instrumental and Pavlovian controllers. Here, we assessed the role of catecholamines in this interplay. We investigated the effects of the catecholamine reuptake inhibitor methylphenidate (MPH) in 100 healthy subjects using a combined appetitive and aversive Pavlovian-to-instrumental transfer (PIT) paradigm, including approach and withdrawal actions. By administering the drug after learning, our design allowed us to establish that MPH can also bias action outside a learning context by directly modulating the interaction of Pavlovian cues with instrumental action. Previously we showed that the effect of MPH on bias varied across these individuals as a function of their working memory (WM) span capacity (Swart et al., 2017). Here, we show by assessing both approach and withdrawal actions that MPH enhanced not only the invigorating effect of appetitive cues on active approach but also the inhibitory effect of appetitive Pavlovian cues on active withdrawal and the invigorating effect of aversive cues on active withdrawal. Thus, in participants with high WM capacity, MPH boosted both approach and withdrawal PIT. Taken together, this pattern of effects is most consistent with the hypothesis that MPH modulates the *cognitive control of Pavlovian biasing* in a baseline-state-dependent manner, in line with the well-established inverted U-shaped relationship between catecholamine receptor stimulation in prefrontal cortex and cognitive control.

## Introduction

Value-based decision making is regulated by a delicate interplay of instrumental and so-called ‘Pavlovian’ controllers. On the one hand, our decisions are shaped by what we learnt from the outcomes of past actions. On the other hand, cues or contexts that promise rewards or threaten something bad may elicit hardwired ‘Pavlovian’ responses. As such, the Pavlovian controller may affect ongoing instrumental responding, a process known as Pavlovian-to-instrumental transfer (PIT). Most of the time these Pavlovian and instrumental controllers concur, so that their interaction is cooperative. For example, a goal-directed approach action to obtain a reward can be enhanced by Pavlovian invigoration of behaviour elicited by the reward cue. In those situations, it can be difficult to disentangle the respective impact of each controller. However, sometimes Pavlovian response tendencies are at odds with optimal, learnt responses, leading to interference with instrumental control. Such Pavlovian interference has been implicated in the aetiology and prognosis of several psychiatric disorders, e.g., addiction, depression, ADHD, and personality disorders (Dayan et al., 2006; Garbusow et al., 2022; Garbusow et al., 2016; Heinz et al., 2016; Huys et al., 2016; Hallquist et al., 2018; Nord et al., 2018; Geurts et al., 2022a; Geurts et al., 2022b; Geurts et al., 2022c). Understanding the neurocognitive mechanisms of PIT is essential to comprehensively understanding and treating these disorders.

The major ascending catecholamine (dopamine and noradrenaline) system represents a particularly good candidate for controlling PIT. In fact, there are at least two routes via which catecholamines may act. First, evidence from studies with experimental animals indicates a key role for *striatal* dopamine in the *generation* of incentive, in particular appetitive, motivational biases of approach behaviour (Berridge and Robinson, 2003; Robbins and Everitt, 2007). For example, appetitive Pavlovian cues elicit striatal dopamine release (Flagel et al., 2011; Wassum et al., 2011), and dopamine depletion (6-OHDA) or augmentation (amphetamine), respectively, attenuate or boost conditioned reinforcement (Taylor and Robbins, 1986; Parkinson et al., 2000). Indeed, in our previous study, we speculated that the catecholamine transporter blocker methylphenidate (MPH) modulated the generation of motivational biases through direct action on striatal dopamine and the associated balance between activity in the direct and indirect basal ganglia pathways (Swart et al., 2017). However, a second, complementary, possibility is that we can understand the effects of MPH in terms of *cortical* dopamine, required to *suppress* the Pavlovian interference with ongoing instrumental responding. This hypothesis is in line with a longstanding literature implicating the cortical catecholamines, in particular prefrontal dopamine, in cognitive control (Cools and D’Esposito, 2011; Ott and Nieder, 2019). Thus, while striatal dopamine can modulate motivational biases, frontal dopamine can attenuate or ‘cognitively control’ such biases (Scholz et al., 2022).

The present study extends previous work and our previous study on MPH’s effects on Pavlovian control (Swart et al., 2017) in several key ways through employing a PIT paradigm that allows us to quantify valence-specific (through a neutral cue baseline) and action-specific (through active and passive approach and withdrawal conditions) PIT (Huys et al., 2011). First, unlike previous studies on dopamine’s role in Pavlovian biasing that indexed biasing using mixed Pavlovian/instrumental learning paradigms (Guitart-Masip et al., 2014; Guitart-Masip et al., 2012; Swart et al., 2017; de Boer et al., 2019; Soutschek et al., 2020; Scholz et al., 2022), instrumental and Pavlovian conditioning are unmixed in the current study and occur prior to the critical PIT phase. This PIT phase thus cleanly isolates the effect of incidental, task-irrelevant Pavlovian cues on instrumental behaviour in extinction, i.e., in the absence of learning. Importantly, this separation of learning and testing Pavlovian bias allowed us to administer MPH only after the instrumental and Pavlovian learning phases. This eliminates the possibility that the observed effects of MPH on Pavlovian bias are due to MPH effects on instrumental and Pavlovian learning, and allows us to isolate MPH’s effect on the transfer of Pavlovian biases on instrumental control (Swart et al., 2017; de Boer et al., 2019). Second, our paradigm allows us to establish the valence specificity of MPH’s effects on Pavlovian conflict control, through the inclusion of a neutral cue, as well as the action specificity of Pavlovian bias, separating passive and active approach and withdrawal actions. These features allowed us to establish whether effects of MPH were restricted to appetitive Pavlovian activation of approach responses, or whether MPH effects extend to aversive biases and aversive actions.

Third, in our previous study, effects of MPH varied greatly across individuals. This individual variability was accounted for by differences in WM capacity. Specifically, MPH attenuated Pavlovian biasing in participants with low baseline WM capacity but enhanced Pavlovian biasing in participants with high baseline WM capacity. Such a pattern is in line with extant evidence for WM-dependent effects of MPH on task performance (e.g. van der Schaaf et al., 2014; Rostami Kandroodi et al., 2021). Nevertheless, replication of this between-subject effect is key, particularly given the recent observation that between-subject brain-phenotype associations are often smaller than expected (Marek et al., 2022).

The results of the present study firmly establish that MPH biases action by modulating the impact of Pavlovian cues on instrumental action depending on individual differences in WM capacity. We show that the effect is also present outside an instrumental learning context when Pavlovian cues are irrelevant to behaviour. This considerably strengthens the conclusion that the catecholamines modulate Pavlovian control. Notably, the results demonstrate that the effect of MPH holds for both appetitive to aversive Pavlovian cues, and for both appetitive instrumental approach and aversive instrumental withdrawal actions. Finally, we replicate that the direction of MPH’s effect depends on individual differences in WM capacity, so that Pavlovian biasing is attenuated in participants with low WM capacity but enhanced in those with high WM capacity after drug administration.

Together, we argue that this pattern of effects mostly accords with the idea that MPH enhances or impairs the *cognitive control of Pavlovian biasing* in a baseline-state-dependent manner, in line with the well-established inverted U-shaped relationship between catecholamine receptor stimulation, likely in prefrontal cortex and cognitive control (Cools and D’Esposito, 2011).

## Results

**Figure 1.**
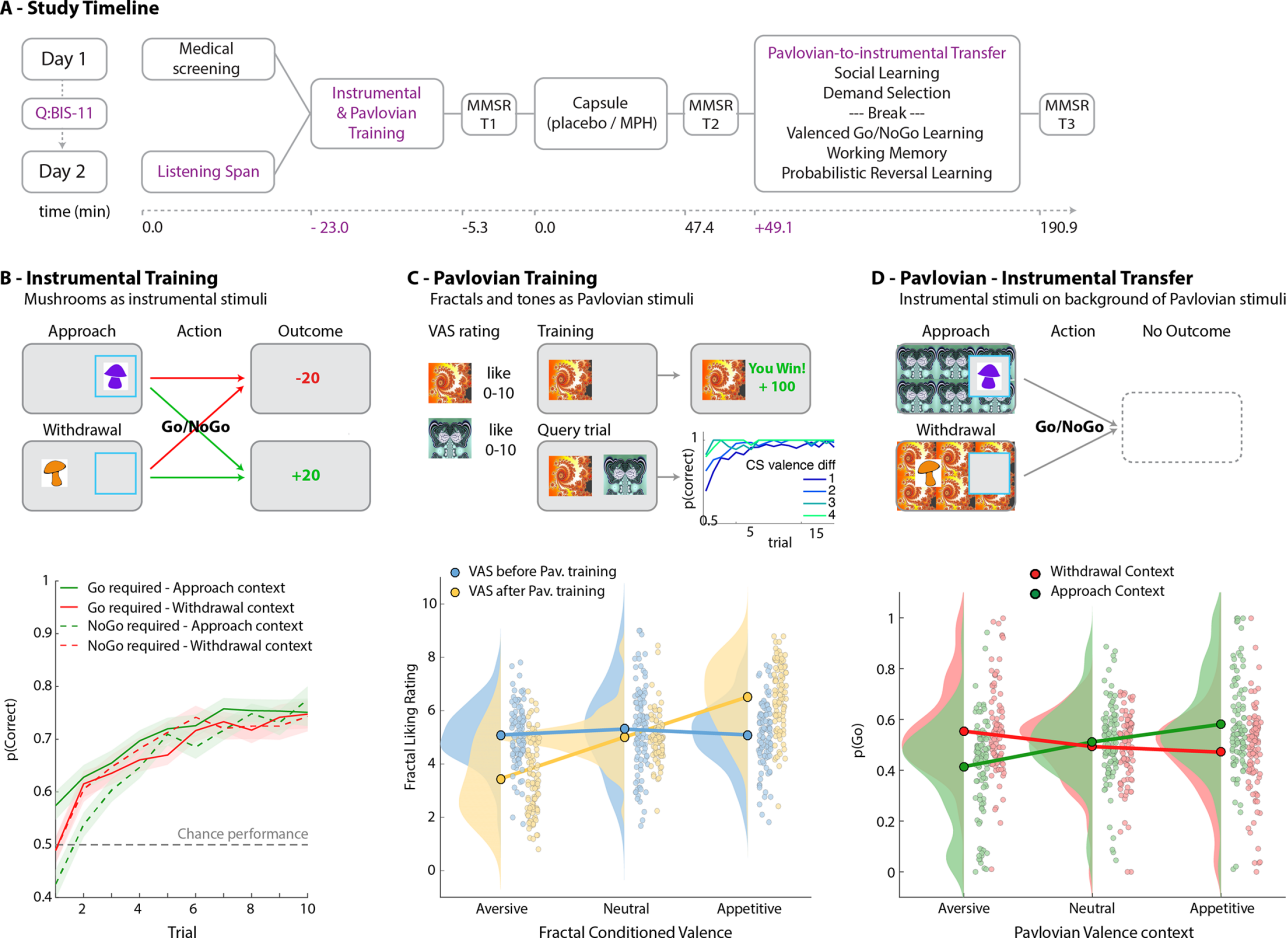
Experimental design and basic results. (**A**) Study timeline. A battery of six tasks was performed in a fixed order on 2 days. The Pavlovian-to-instrumental transfer (PIT) task was always performed first to minimise interference between training and transfer. Average timings are indicated, with timings most relevant for the PIT task data presented here in purple. Tasks marked in black have been published/are in preparation elsewhere (Swart et al., 2017; Froböse et al., 2018; Cook et al., 2019; Rostami Kandroodi et al., 2021). Working memory (WM) was assessed with a listening span task prior to drug intake on day 2 of the study. Impulsivity was assessed with the Barratt Impulsiveness Scale (BIS-11), which participants filled out in the interval between the two testing sessions like the other Mood & Medical Symptom ratings (MMSR). (**B–D**) PIT task design and main task effects (averaged across drug conditions) for the three task phases. (**B**) Instrumental training. Design (upper panel): Participants had to choose whether to click (‘Go’) or not click (‘NoGo’) with the mouse inside the blue frame. Trials were grouped into Approach and Withdrawal blocks. In the Approach context, a Go response resulted in ‘collecting’ the mushroom, while NoGo meant not collecting it. In the Withdrawal context, a Go would ‘discard’ the mushroom, while NoGo would keep it. Each mushroom only appeared in either Approach or Withdrawal contexts. For each mushroom, one action (Go or NoGo) was correct and rewarded with 75% probability. The other action was punished with 75% probability. Participants were instructed that the outcomes received counted towards a bonus payment. Each block contained six unique mushrooms. Results (lower panel): mean ± standard error of the mean. Participants learnt to make the correct response for each of the required actions and Action Contexts. Note that for approach contexts, people initially displayed a Go bias, which led to above chance performance for Go-Approach cues and below-chance performance for NoGo-Approach cues. However, at the end of training, all cue/context combinations reached the same plateau, which approximately probability-matched the reward contingency. (**C**) Pavlovian conditioning. Design (upper panel): In the conditioning trials, five fractals were presented repeatedly, followed deterministically by monetary outcomes of five value levels: high (100) or low (10) reward, nothing (0), low (–10), or high (–100) punishment. Intermixed with the conditioning trials, 18 query trials were presented, where participants had to select the most rewarding of a random selection of two of the stimuli. The line plot shows average probability of selecting the higher conditioned stimulus (CS) across participants and drug sessions, as a function of valence level difference. Participants rapidly learned to select the better CS. Prior to and post conditioning, subjects were asked to rate how much they liked each fractal on a visual analogue scale (VAS). Results (lower panel): VAS significantly increased for appetitive CSs, while they were reduced for aversive stimuli. Circles represent individual subjects. (**D**) PIT. Design (upper panel): Participants performed the instrumental training task in nominal extinction (they were instructed that their actions still counted towards their payment). On every trial, one of the Pavlovian CSs tiled the background. Results (lower panel): Proportion of Go responses independent of required response. In the Approach context (green), presence of a positive Pavlovian CS increased Go (active approach), while a negative Pavlovian CS reduced Go (relative to a neutral CS). In contrast, in the Withdrawal context, the negative Pavlovian CS enhanced Go (active withdrawal), while the positive CS reduced Go. For panels C and D, results are collapsed within valence (i.e. high and low reward CS and high and low punishment CS were averaged). See Figure 1—figure supplement 1 for each of the valence levels separately.

In a double-blind, placebo-controlled, cross-over design, we assessed the effect of MPH on motivational control using a previously established PIT task (Huys et al., 2011; Geurts et al., 2013) in 100 healthy subjects (see Methods). In this paradigm, subjects are first trained instrumentally to either approach or withdraw from a cue (different coloured mushrooms, Figure 1B), in order to maximise reward and minimise loss. This approach/avoidance may be either active (taking action to approach or avoid the cue) or passive (withholding responses will lead to approach/avoidance of the cue). Subjects then performed this task in the context of a Pavlovian conditioned stimulus (CS; fractals paired with reward or punishment; Figure 1C) to assess how these cues affect ongoing instrumental behaviour. Under placebo (PLA)/baseline conditions, humans typically display valence- and action-specific PIT effects, such that an appetitive Pavlovian context enhances active approach but reduces active withdrawal, while an aversive Pavlovian context reduces active approach but enhances active withdrawal (Huys et al., 2011). Critically, we administered the dopamine/noradrenaline transporter blocker MPH (Ritalin) after learning but prior to transfer to investigate the action and/or valence specificity of catecholaminergic effects on PIT in the absence of learning. Moreover, we assessed WM with a listening span task before drug intake on day 2 of the experiment and impulsivity with the Barratt Impulsiveness Scale (BIS-11) before day 2 (Figure 1A).

**Figure 1—figure supplement 1.**
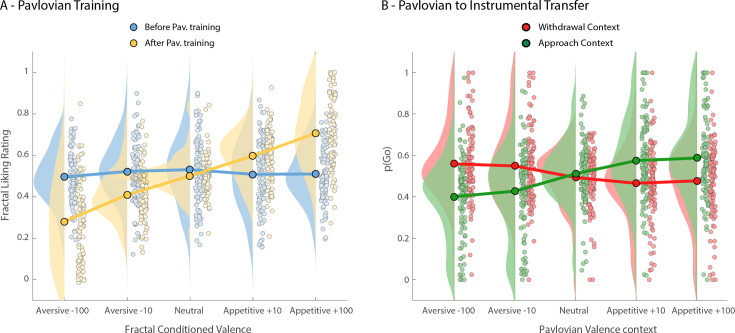
Basic VAS and PIT results for five levels of conditioned stimulus reinforcement. (**A**) Pavlovian conditioning effects and Pavlovian-to-instrumental transfer (PIT) effects as a function of all five levels of conditioned stimulus (CS) reinforcement. Liking ratings scale linearly with the levels of reinforcement, i.e., the CS associated with a large reward was liked more than the CS associated with a small reward, and vice versa for punishments. (**B**) In contrast, PIT effects are a function of the sign (aversive, neutral, appetitive), but not the magnitude (10 vs. 100), of the CS valence. In other words, PIT effects were equally strong for large and small reinforcers of each valence.

We predicted a classic PIT effect under placebo: Appetitive conditioned Pavlovian cues would promote approach (Go) responses, encouraging instrumental approach and impairing instrumental withdrawal actions. Aversively conditioned Pavlovian cues, however, should promote withdrawal, impairing instrumental approach and encouraging instrumental withdrawal actions. In line with previous work from the same participant cohort (Swart et al., 2017; Rostami Kandroodi et al., 2021), we predicted baseline-dependent effects of MPH on this classic PIT effect, and we interrogated the specific effects of MPH to gain insight into the underlying mechanisms.

More specifically, based on the primarily striatal bias-modulation account, one would expect MPH to have a disproportionate effect on global behavioural *activation* by appetitive Pavlovian cues. In contrast, the cognitive control account would predict a more general effect, such that MPH affects both appetitive and aversive Pavlovian biases and both appetitive and aversive instrumental behaviours.

### Action-specific PIT

First, basic task effects from previous studies were replicated. Subjects showed strong action-specific PIT across drug conditions (Action Context × Valence Context: X^2^=15.4, p<0.001, Figure 1D). In the Approach context, i.e., when an approach was the available active response, subjects made more Go responses in the presence of an appetitive CS (X^2^=8.2, p=0.004), and fewer Go responses in the presence of an aversive CS (X^2^=9.8, p=0.002), relative to a neutral CS (Main effect of Valence Context in Approach context: X^2^=10.1, p=0.001). On the contrary, in the Withdrawal context, i.e., when the available active response was withdrawal, subjects made more Go responses in the presence of an aversive CS (X^2^=10.4, p=0.001), but not in the presence of an appetitive CS (X^2^=0.7, p=0.4), relative to a neutral CS (Main effect of Valence Context in Withdrawal context: X^2^=7.5, p=0.006). In addition, there was a main effect of Valence Context, independent of Action Context, such that the presence of an aversive CS had an inhibitory influence, while an appetitive CS had an invigorating effect on Go responses (Valence Context: X^2^=10.2, p=0.001).

### WM span explains individual differences in MPH-induced changes in PIT

**Figure 2.**
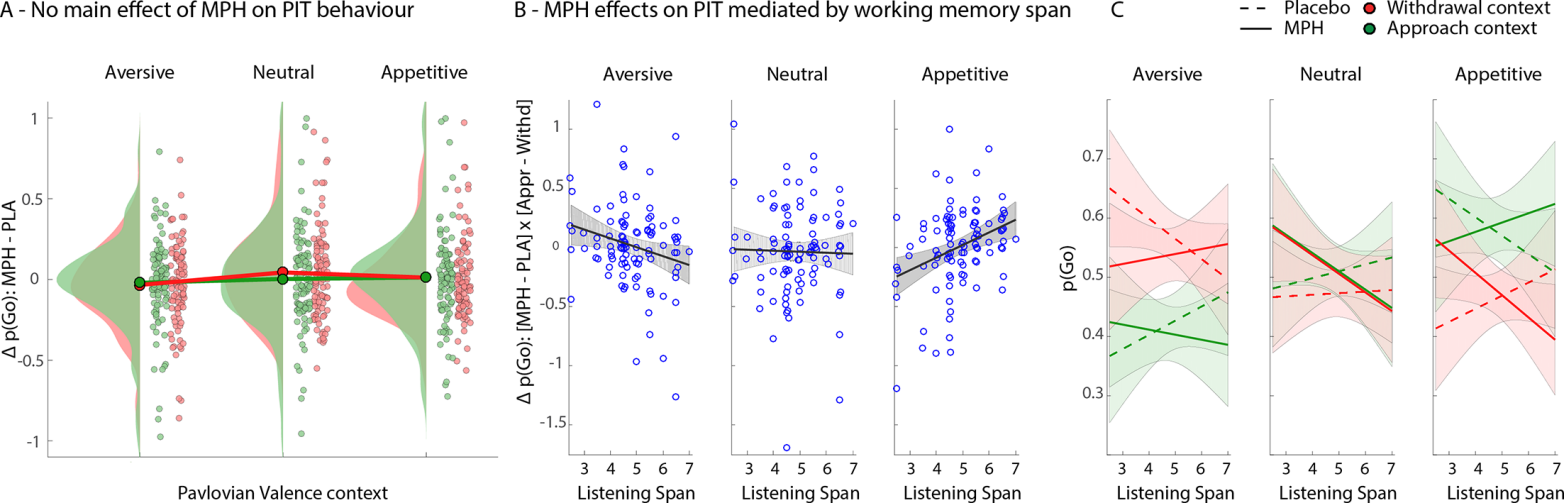
Effects of methylphenidate (MPH) on Pavlovian-instrumental transfer (PIT). (**A**) Individual data points plus associated density distributions for the difference in likelihood of a Go response under MPH minus placebo (PLA). Data are plotted as a function of Action and Valence Context. There was no main effect of MPH. (**B, C**) MPH affects action-specific PIT (i.e. the Action Context-specific impact of Pavlovian valence on invigoration) as a function of baseline working memory capacity. Plots show the regression line and 95% confidence intervals, and in B also show individual data points. (**B**) Effects of MPH on action-specific PIT (Action Context × Valence Context), for each Pavlovian Valence Context (Action Context × Valence Context × Drug × Listening Span: X^2^=9.5, p=0.002) (see figure supplement for each of the five valence levels separately) (**C**) Breaking down the four-way interaction demonstrates the full reversal of the effect of drug on action-specific PIT as a function of working memory (listening span) performance. In short, under placebo, action-specific PIT is present in people with low, but not high, working memory span, while under MPH action-specific PIT is present in people with high, but not low, working memory span. See Figure 2 - Figure supplement 1 for each of the valence levels separately.

Our main finding is that action-specific PIT was modulated by MPH as a function of WM capacity, as measured by the Listening Span Task (Action Context × Valence Context × Drug × Listening Span: X^2^=9.5, p=0.002; Figure 2B) without a main effect of MPH on PIT (Action Context × Valence Context × Drug × Listening Span: X^2^ <1, p=0.99; Figure 2A). To interpret this interaction, we followed up with a tertile split based on WM span. In brief, low-span subjects (lowest tertile) showed action-specific PIT under placebo (X^2^=9.6, p=0.002), but not under MPH (X^2^=2.4, p=0.12). Conversely, high-span subjects (highest tertile) showed action-specific PIT under MPH (X^2^=7.4, p=0.007; Figure 2C), but not under placebo (X^2^=2.8, p=0.10). Furthermore, breakdown of this four-way interaction into its component simple three-way interactions (Table 1) revealed significant effects of MPH in both appetitive and aversive Valence Contexts and not for the neutral context. Based on current understanding of striatal dopamine’s role in appetitive activation (Berridge and Robinson, 1998; Robbins and Everitt, 2007; Schultz, 2011), one might have expected effects of MPH especially on the appetitive activation of approach actions. Moreover, in high-span subjects, MPH also increased the *inhibitory* effects of *appetitive* Pavlovian cues, as well as the *activating* effects of *aversive* Pavlovian cues on withdrawal actions. Note, however, that the simple interaction for withdrawal only is much less significant than the four-way interaction, including the approach context, and that the effects in the approach condition are opposite to withdrawal (Figure 2C), so these post hoc simple interactions should be interpreted with caution.

**Figure 2—figure supplement 1.**
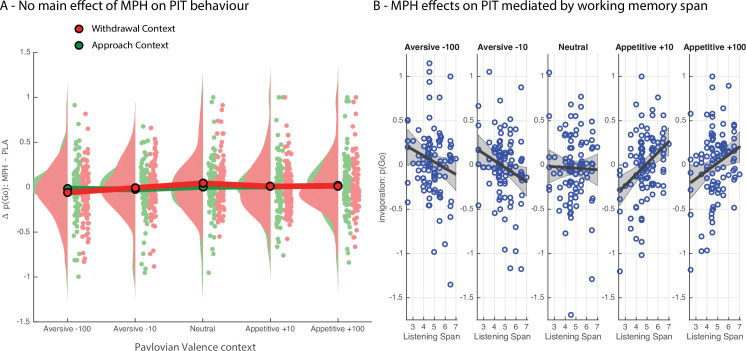
Effects of methylphenidate (MPH) on Pavlovian-instrumental transfer (PIT). (**A**) Effects of methylphenidate on Pavlovian-to-instrumental transfer (PIT) as a function of all five CS valence levels, which again are a function of sign but not valence.

**Table 1.** Breaking down the 4-day interaction into component factors; PLA: placebo; MPH: methylphenidate; WM: working memory.

	X^2^	p-Value
**Main four-way interaction**		
Action Context × Valence Context × Drug × WM span	9.5	0.002
**Simple interaction: Valence Context**		
Appetitive: Action Context × Drug × WM span	9.4	0.002
Neutral: Action Context × Drug × WM span	0.2	0.7
Aversive: Action Context × Drug × WM span	5.0	0.026
**Simple interaction: Action Context**		
Approach: Valence Context × Drug × WM span	1.2	0.3
Withdrawal: Valence Context × Drug × WM span	5.9	0.015
**Simple interaction: Drug**		
MPH: Action Context × Valence Context × WM span	0.99	0.3
PLA: Action Context × Valence Context × WM span	2.1	0.15

In a follow-up analysis, we established that the magnitude of the PIT effect was not significantly different between the aversive and the appetitive CS (reverse-coded aversive context, Drug × Action Context × Listening Span × Valence Context (appetitive/aversive): X^2^=0.2, p=0.70).

## Discussion

The current study demonstrates that MPH modulates the differential impact of Pavlovian biases on instrumental approach and withdrawal actions, depending on individual differences in WM capacity. Specifically, MPH reduced Pavlovian biasing in participants with low WM capacity but increased it in those with high WM capacity. These findings reinforce and refine the interpretation of our previous observation that MPH alters Pavlovian biasing of both instrumental learning and choice, depending on WM capacity (Swart et al., 2017). Unlike that previous study, the MPH effects in the current study cannot reflect modulation of biased learning, but must reflect the expression, or transfer, of Pavlovian biases onto action. This is because MPH was administered *after* the instrumental and Pavlovian conditioning phases were completed. Moreover, the effects of MPH were present across valence and action dimensions, extending to aversive Pavlovian conditions and withdrawal actions. Together these results indicate that catecholamine enhancement alters motivational biases of behaviour.

### Advances over prior work

The present study adds value over and above previous studies in the following key ways. It establishes that MPH changes the influence of Pavlovian cues on instrumental action in a manner that is independent of any effects on instrumental and/or Pavlovian learning. Previous work did not dissociate motivational biases in choice (Pavlovian) and biases in instrumental learning (Guitart-Masip et al., 2012; Guitart-Masip et al., 2014; Scholz et al., 2022) or relied on teasing these apart through computational modelling (Swart et al., 2017). Here, in contrast, this distinction is ascertained by the experimental design: Instrumental and Pavlovian conditioning preceded the interaction between Pavlovian and instrumental stimuli in the separate transfer phase. Importantly, this transfer phase was conducted in nominal extinction, which precludes effects of MPH influencing PIT via outcome learning. Thus, these findings triangulate our previous findings (Swart et al., 2017) within the same subject sample, evidencing their robustness, and show that the effects of MPH can indeed, by experimental design, be attributed to Pavlovian biases specifically.

The current study conceptually replicates our previously reported effects of MPH on Pavlovian choice bias (Swart et al., 2017). Crucially, however, through the valence and action specificity of the design, the current study revealed that MPH affects both appetitive and aversive Pavlovian biases in both appetitive and aversive instrumental behaviours. The generality of the effect of MPH on PIT, particularly across action domains, shows that the influence of MPH on Pavlovian biases is more than a simple modulation of behavioural invigoration driven by reward or punishment cues.

Below, we consider three explanatory accounts of our findings: a primarily striatal account, a primarily cortical account, and a cortico-striatal balance account. These correspond, respectively, to mechanisms of striatal bias generation, frontal cognitive control, and the dynamic interplay between the two. Our results appear inconsistent with the striatal bias generation account alone, whereas both the cortical and balance accounts remain plausible explanations and warrant further investigation in future studies.

### Global modulation of Pavlovian biases by MPH challenges a striatal bias generation account

In our previous study (Swart et al., 2017) we attributed the effect of MPH to a modulation of striatal dopamine and associated shifts in the balance of activity between the direct and indirect pathway of the basal ganglia. Specifically, in Swart et al., we proposed that the coupling of striatal architecture of D1 and D2 pathways with behaviour might parsimoniously explain the effect of MPH on Pavlovian biases. Indeed, MPH increases striatal dopamine availability (Volkow et al., 2002; Clatworthy et al., 2009). However, Pavlovian cues have been shown to affect striatal dopamine in a valence-dependent manner. Appetitive Pavlovian cues elicit peaks and aversive Pavlovian cues elicit dips in dopamine release (Tobler et al., 2005; Day et al., 2007; Matsumoto and Hikosaka, 2009; Cohen et al., 2012). Increases in striatal dopamine levels have been related to activation of the ‘Go’ pathway mainly relying on D1 receptors (Hernández-López et al., 1997) and promoting behavioural activation (Mink and Thach, 1991; DeLong and Wichmann, 2007). Decreases, on the other hand, activate the ‘NoGo’ pathway (Hernandez-Lopez et al., 2000), promoting behavioural inhibition. From this model, one would expect MPH to have a disproportionate effect on global behavioural *activation* by appetitive Pavlovian cues.

By contrast, when assessing the action-specific nature of behavioural activation (i.e. approach vs. withdrawal), the present study revealed that MPH also enhanced the *inhibitory* effects of appetitive Pavlovian cues on *active* withdrawal, as well as the enhancing effects of aversive Pavlovian cues on active withdrawal. Specifically, in participants with high WM capacity, MPH boosted both approach and withdrawal PIT. Thus, while effects in the approach domain were in the expected direction, with MPH boosting the invigorating impact of appetitive cues and the *suppressive* impact of aversive cues on approach Go actions, this direction was reversed for withdrawal-prompted Go actions. Here, MPH boosted the *suppressive* impact of appetitive cues and the *invigorating* impact of aversive cues on withdrawal Go actions.

### Global modulation of Pavlovian biases by MPH supports a frontal cognitive-control account

An alternative account of the observed global modulation of PIT by MPH is that the drug may modulate the ability to exert top-down cognitive control over any type of ‘hardwired’ Pavlovian bias. This account is in line with our recent results, demonstrating that administration of the COMT inhibitor tolcapone, which increases prefrontal but not striatal dopamine, also attenuates Pavlovian biases in a simpler Go/NoGo learning task (Scholz et al., 2022). This cognitive control account is substantiated by the pattern of MPH effects as a function of WM capacity. This pattern is in accordance not only with the WM-dependent pattern of MPH effects observed in our own previous study (Swart et al., 2017) but also with previously reported effects of a D1 receptor agonist on appetitive PIT (Soutschek et al., 2020). Soutschek et al., 2020, showed that administration of a high dose of a D1 receptor agonist increased PIT to a greater degree in participants with higher WM capacity. We have known for a long time that there is an optimum level of dopamine receptor stimulation in prefrontal cortex, where both too little and too much prefrontal dopamine is detrimental for cognitive control and WM (Williams and Goldman-Rakic, 1995; Zahrt et al., 1997; Arnsten, 1998; Cools and D’Esposito, 2011). Therefore, the current pattern of effects might reflect MPH-induced enhancement of cognitive control of Pavlovian bias in participants with low WM capacity, but MPH-induced impairment of cognitive control of Pavlovian bias in participants with high WM capacity.

There are various mechanisms by which MPH might have boosted the control of Pavlovian biases in low-span subjects. MPH might enhance the value of cognitive (instrumental) effort by enhancing the weights on the benefits of effort (Shenhav et al., 2013; Shenhav et al., 2017; Westbrook et al., 2020) and/or improved the inhibition of automatic, prepotent response (Scheres et al., 2003) by promoting the frontal drive of the subthalamic nucleus, thus raising decision thresholds (Cavanagh et al., 2011). It might also have increased the subjective estimation of the controllability of instrumental actions by acting on rostral medial frontal cortex (Duan et al., 2021; Ligneul et al., 2022). In any case, given the implication of the medial frontal cortex in the control of Pavlovian biasing (Cavanagh and Frank, 2014), MPH may have acted on the frontal cortex of low-span subjects to attenuate Pavlovian bias. Under this cognitive control hypothesis, the observed increase in Pavlovian bias in high-span subjects may reflect overdosing of relatively high baseline levels of dopamine in prefrontal cortex. This hypothesis concurs with the well-established observation that excessive dopamine receptor stimulation in the prefrontal cortex can undermine prefrontal function by quelling neural activity via blockade of glutamatergic input and potentiation of GABAergic interneuron activity (Zahrt et al., 1997; Seamans and Yang, 2004; Cools and D’Esposito, 2011).

### Global modulation of Pavlovian biases by MPH might arise from an altered balance of striatal and frontal control

A final alternative hypothesis is that the effects of MPH do not have a purely striatal (bias generation) or frontal (bias control) origin, but rather that MPH may shift the balance between these two. WM capacity may then provide an index of the relative balance of the influence of Pavlovian subcortical ‘drivers’ (ventral striatal/amygdala; Cardinal et al., 2002) vs. instrumental, cognitive (dorsal fronto-striatal) control systems in shaping behaviour. The ‘baseline state’ of this balance may determine the net effect of MPH, which acts both in the striatum by blocking the dopamine transporter and in the frontal cortex by blocking the noradrenaline transporter, enhancing particularly those systems that, at baseline, have the least ‘weight’ on behaviour. Thus, in participants with low WM capacity (putatively associated with reduced frontal executive function and cognitive control), MPH may primarily enhance frontal suppression of Pavlovian bias. By contrast, in participants with high WM capacity, MPH may primarily boost the actions of the ventral striatum (driving Pavlovian bias).

### Limitations and future studies

With respect to limitations, we acknowledge that we focused throughout this paper on dopamine, although there might be a strong contribution of MPH’s modulation of noradrenaline to our results. Noradrenaline has been implicated in aversive PIT (Campese et al., 2017) and more broadly, in regulating reward- and aversion-driven behaviour (via the medial prefrontal cortex) in concert with the dopaminergic system (Pastor and Medina, 2021). To isolate the role of striatal dopamine, in future studies, effects of MPH should be compared, for example, with those of the noradrenaline transporter blocker atomoxetine, which modulates cortical catecholamines, but not striatal dopamine (Bymaster et al., 2002). Moreover, our main result, although causally impacted by MPH, relies on a correlation with WM capacity, which precludes causal inference. Future PIT experiments might further these results by experimentally modulating WM load during the preceding instrumental learning phase.

### Summary

In summary, our results clearly demonstrate that catecholamines shape Pavlovian influences on instrumental behaviour, in an action-specific manner. Future pharmacological and chemical neuroimaging work is required to definitively disentangle the different (not mutually exclusive) cognitive control and bias generation accounts of MPH-related boosting of motivational biasing of instrumental actions.

## Methods

### General procedure and pharmacological intervention

The study consisted of two test sessions with an interval of 1 week to 2 months. The first test day started with informed consent, followed by a medical screening. Participation was discontinued if subjects met any of the exclusion criteria (Appendix – *Method 1*). On both test days, subjects first completed baseline measures, as well as the Instrumental and Pavlovian phases of the PIT task (for details, see below in Task design). Subjects then received a capsule containing either 20 mg MPH (Ritalin, Novartis) or placebo, in a double-blind, placebo-controlled, cross-over design. MPH blocks the dopamine and noradrenaline transporters, thereby diminishing the reuptake of catecholamines. When administered orally, MPH has a maximal plasma concentration after 2 hr and a plasma half-life of 2–3 hr (Kimko et al., 1999). Below we denote capsule intake as t=0.

As described above, the PIT training phases were started prior to capsule intake, starting at t = −23.0 (±2.3) min. The transfer phase of the PIT task battery was the first task subjects completed following intake, to reduce interference (t=49.1 ± 2.1 min) post intake, which is well within the peak of plasma concentration. This task was followed by five other tasks (Figure 1) published elsewhere (Swart et al., 2017; Froböse et al., 2018; Cook et al., 2019; Rostami Kandroodi et al., 2021). Both test days lasted approximately 4.5 hr, which subjects started at the same time of day (maximum difference of 45 min). Blood pressure, mood, and potential medical symptoms were monitored three times each day: before capsule intake (t=−5.3 ± 1.7 min), directly prior to start of the task battery (t=47.4 ± 7.6) and after finishing the task battery (190.9±7.9). Subjects were instructed to abstain from alcohol and recreational drugs 24 hr prior to testing and from smoking and drinking coffee on the days of testing. Subjects completed self-report questionnaires at home between (but not on) test days. Upon completion of the study, subjects received a monetary reimbursement or study credits for participation. The study was in line with the local ethical guidelines approved by the local ethics committee (METC Oost Nederland: protocol NL47166.091.13), pre-registered (trial register NTR4653), and in accordance with the Helsinki Declaration of 1975. Data and code for the study are freely available at https://doi.org/10.34973/8m6j-9695.

### Participants

106 healthy, young adults participated in this study and were recruited via flyers around the campus and the digital participant pool of the Radboud University, Nijmegen. All participants were native Dutch speakers and provided written informed consent to participate in the study. Exclusion criteria comprised a history of psychiatric, neurological, or endocrine disorders. *Appendix – Method 1* presents a complete overview of the exclusion criteria. Data from two participants were not available due to technical problems. Furthermore, data from four participants were incomplete due to medical (mild arrhythmia: n=1, elevated heart rate and nausea: n=1) problems and drop-outs (n=2). Thus, the analyses include 100 adult participants (aged 18–28 years, mean age 21.6, SD = 2.3, 54 women, 80 right-handed), where 48 participants received MPH on the first testing session. Additional demographic information and results from baseline neuropsychological assessment and self-report questionnaires of included participants are reported in Table 2. Two participants had trouble swallowing the capsule such that for one participant the capsule dissolved orally before swallowing and for the other participant content of the capsule was dissolved in water.

**Table 2.** Demographics, experimental information and results of neuropsychological assessments and self-report questionnaires at baseline.

N=100	Characteristic	Measure	Mean (std)	Min-max	Range*
**Demographics**	Age	Years	21.6 (2.3)	18–28	–
	Gender	Men/women	50/50	–	–
**Experimental information**	Order	Placebo first/MPH first	52/48	–	–
	Mean delay methylphenidate to task start	Minutes	49.1 (2.1)	40–61	–
**Neuropsychological assessment**	Verbal intelligence	NLV	93.5 (7.7)	75–114	55–145
	Working memory capacity	Listening span: total span	4.8 (1.1)	2.5–7	0–7
		Digit span^†^ Forward Backward	16.8 (3.6)	10–26	0–28
			14.4 (3.1)	8–23	0–28
**Self-report questionnaires**	Impulsivity	BIS-11: total score	62.0 (8.5)	43–93	30–120
	Need for Cognition	NCS	63.3 (10.5)	38–82	18–90
	Depressive symptoms	BDI	3.6 (3.9)	0–21	0–63
	Behavioural activation	BAS: total score	23.3 (4.0)	15–34	13–52
	Behavioural inhibition	BIS	16.3 (3.6)	7–23	7–28
	Anxiety symptoms	STAI	32.5 (6.9)	23–55	20–80
	Social support	MDSPSS: total	70.5 (9.6)	43–84	12–84
	Social status	BSMSS: total	48.0 (12.7)	14.5–66	8–66
	Social dominance	SADQ: social	4.1 (0.9)	2.1–5.9	1–7
	Aggressive dominance	SADQ: aggressive	2.6 (0.6)	1.3–4.6	1–7

Demographic and background characteristics of participants included in the analysis. Questionnaires included the Beck Depression Inventory (BDI; Beck et al., 1996), Behavioral Inhibition Scale/Behavioral Activation Scale (BIS/BAS; Carver and White, 1994), Spielberger Trait Anxiety Inventory (STAI; Spielberger et al., 1983), Multidimensional Scale of Perceived Social Support (MDSPSS; Zimet et al., 1988), Social and Aggressive Dominance Questionnaire (SADQ; Kalma et al., 1993), and Barratt Simplified Measure of Social Status (BSMSS; Barratt, 2006).

* Range reflects the possible range of scores on the questionnaires, whereas min-max and mean (std) reflect the participant data.

† Average across two testing days. Digit span was completed prior to drug intake.

### Task design

We used the task as previously described in Huys et al., 2011. In short, the task was divided into two blocks (approach and withdrawal), each consisting of an instrumental training and a Pavlovian training, which were both completed prior to medication (or placebo) intake, and a PIT stage (Figure 1).

### Instrumental training

The instrumental task (Figure 1B) was an approach or withdrawal Go/NoGo learning task, framed in terms of collecting or discarding mushrooms. In the approach block, participants chose whether to collect the mushroom by moving the mouse towards and clicking on the stimulus (approach-Go) within a response window of 1.5 s or not collect the mushroom by abstaining from a response for 1.5 s (approach-NoGo). In the withdrawal block, participants chose whether to discard mushrooms by clicking in a blue frame located on the opposite side of the stimulus (withdrawal-Go) or do nothing (withdrawal-NoGo). The outcome (±20 Euro cents) was then presented in the middle of the screen. Reinforcements were probabilistic, with the ‘correct’ response for each mushroom leading to gain or avoidance of loss on 75% of the trials. Correct trials were those on which participants discarded a ‘bad’ or kept a ‘good’ mushroom, and those on which they collected a ‘good’ or refrained from collecting a ‘bad’ mushroom. Participants had to learn the better response for each stimulus from the noisy reinforcement feedback. There were three ‘good’ and three ‘bad’ mushrooms in each context, meaning that all actions (i.e. approach-Go, approach-NoGo, withdrawal-Go, and withdrawal-NoGo) could be followed by both rewards and punishments. Thus, the expected value of correct approach and withdrawal actions was equal and positive on average.

### Pavlovian training

The second part of the task consisted of a separate classical conditioning procedure. Five compound Pavlovian stimuli (CS), consisting of a fractal visual stimulus (Figure 1C) and a tone, were deterministically paired with five levels of monetary outcomes of [+100, +10, 0, –10, –100]. To ensure that participants paid attention, a query trial was presented on every fifth trial on which two of the five Pavlovian stimuli were presented. Participants then had to choose the highest valued Pavlovian stimuli (Figure 1C) in extinction.

### Pavlovian-to-instrumental transfer

Our central question was whether the impact of Pavlovian Valence Context on instrumental responding is altered by MPH. Therefore, the PIT phase alone was completed 49 min (see above) after drug intake and approximately 72 min after completion of the Pavlovian conditioning phase. In the intervening period, the participants completed mood and medical symptom questionnaires but performed no other tasks. In this transfer phase, subjects chose whether to collect or discard mushrooms while the Pavlovian stimuli tiled the entire background (Figure 1D). Critically, no outcomes were presented. Participants were instructed to continue performing the instrumental task and that their choices would still count towards their final earnings. Regarding the Pavlovian stimuli, participants received no instructions.

### Control analyses

#### Covariates of no interest

We assessed the impact of a number of potential confound factors, using a model comparison approach employing the *anova* function in R. Specifically, we tested whether the following factors improved model evidence by assessing differences in Bayesian information criterion (BIC): Drug testing Order (PLA vs. MPH on day 1), Gender and DRT (a measure for verbal intelligence).

#### Deterministic PIT responding

In past studies using the same task, we and others have observed that sometimes participants respond deterministically to a particular Pavlovian/Action Context combination regardless of the instrumental stimulus presented (e.g. Chen et al., 2023). For example, a participant would always make a Go response when a certain appetitive Pavlovian cue was present in an approach context. Such deterministic responding may reflect a misunderstanding of the task. We therefore repeated our main analysis of interest, excluding all participants who responded deterministically for at least one valence cue/Action Context combination on at least one of the testing days. Note that this is a very conservative approach as this means we excluded anyone who responded deterministically to even 1 out of 20 cue-context combinations.

#### Instrumental training

We analysed instrumental training data for three main reasons. First, to establish that instrumental responses were well learnt. Second, to assess whether the learned responding at the end of the training phase generalised to behaviour in the PIT phase. Third, to be able account for any potential difference in the degree of instrumental conditioning for different instrumental stimuli and subjects during the PIT phase. To assess instrumental learning, we used a logistic mixed effects model, modelling the likelihood of making a correct response as a function of trial number (for each stimulus). To assess whether performance was similar across conditions at the end of learning, we repeated this analysis including only the final five trials of each stimulus. For completeness, these models further included the following factors: Day, Action Context, Listening Span and Impulsivity, Stimulus type (Go vs. NoGo). Next, to assess whether learned instrumental behaviour is successfully retrieved during the PIT stage, and whether the degree of instrumental performance interacted with our effects of interest, we included the final-stage choice behaviour (as an index of the degree of learning) as a covariate to the main analysis of interest. This metric was defined as the proportion of Go responses during the final five presentations of each stimulus during the instrumental training. A putative interaction between the degree of learning and the effect of MPH on PIT behaviour could index a drug-induced change in post-learning consolidation/retrieval, rather than a PIT effect per se. Results of the above analyses are reported in *Appendix – Result 2*.

#### Pavlovian conditioning

As for the instrumental training phase, Pavlovian conditioning was completed prior to drug administration (c.f. Figure 1A). We assessed whether Pavlovian conditioning was successful in two ways. First, we assessed performance (correct/incorrect) on the query trials. We recoded choices as ‘correct’ when participants chose the image associated with a higher number of points and ‘incorrect’ otherwise, and analysed whether during the course of learning, choices were more likely to be correct. This was implemented using a logistic mixed effects model with the covariate trial number and the difference in valence level (1–4). The latter allowed us to assess whether relative values of the different CS were learnt well. Second, we measured explicit valuation of the Pavlovian cues by assessing the change in VAS scores pre-post conditioning as a function of Valence, using a general linear mixed model with the main factors of interest of Valence and Time (pre-conditioning vs. post-PIT). For completeness, these models further included the following factors (reported in *Appendix – Result 2*): Day, Listening Span, and Impulsivity.

Detailed procedures for the WM (listening span test) and impulsivity (Barratt Impulsiveness Scale) assessments are reported in *Appendix – Method 2* and were identical to Swart et al., 2017.

### Statistical analyses

#### CS valence coding

We first performed an analysis to assess whether the data was better captured by a model that coded three valence levels (positive, neutral, negative, i.e. averaging across the high and low conditions within each Valence Context) or by a model that each of the five levels of Valence Context separately (cf. Huys et al., 2011; Geurts et al., 2013). Given that the three-level valence model showed better model fit than the five-level valence model, as indicated by lower BIC and AIC values and a likelihood-ratio test favouring the more parsimonious model, all analyses presented below are based on the three-level CS valence model.Note that in the Appendix – Result 1 we report the five-level analyses also, and main findings do not change.

#### Effects of MPH on PIT

To assess the influence of motivational cues on instrumental behaviour, we analysed invigoration (Go vs. NoGo responding) during PIT. To account for both between- and within-subject variability, these data were analysed with logistic mixed-level models using the lme4 package in R (Bates et al., 2014). Reflecting our objectives, the mixed models included the within-subject factors Drug (MPH vs. placebo), Action Context (approach vs. withdrawal), and Valence Context (appetitive/neutral/aversive), and the between-subject factors Listening Span and Impulsivity. Models included all main effects and interactions, except for the interactions between Listening Span and Impulsivity. All models contained a full random effects structure (Barr et al., 2013). To assess whether any putative effects of MPH on valence context-dependent effects were larger for appetitive than for aversive contexts, we conducted a follow-up analysis where aversive context responses were reverse-coded, leaving out the neutral condition. We report effects significant at an alpha-level of <0.05. Any significant higher-order interactions will be analysed using post hoc simple interactions for ease of interpretation.

#### Control analyses

In the *Appendix – Result 2*, we present a number of control analyses that confirm the robustness of the results: we establish (i) lack of effect of covariates of no interest, including drug administration order, gender, intelligence; (ii) robustness of the results to exclusion of participants with deterministic PIT responding; (iii) successful instrumental conditioning, where we also confirm that the degree of instrumental conditioning did not affect the effects of MPH; and (iv) successful Pavlovian conditioning. In addition, we report results from the Mood & Medical Symptom ratings performed three times on each testing session (*Appendix – Result 3*).

## Data Availability

Individual-level raw and processed data associated with this study cannot be made publicly available because they consist of pseudonymised sensitive human participant data and cannot be considered fully anonymised. Public sharing of these data would therefore not be compatible with applicable privacy legislation/GDPR requirements and Radboudumc data-sharing policy. The data are available through restricted access via the Radboud Data Repository. Researchers wishing to access the data should submit a request through the repository/data access procedure at https://doi.org/10.34973/8m6j-9695. Requests will be assessed by Radboudumc/RDR in accordance with ethical approval, informed consent, privacy requirements, and the proposed scientific use of the data. Access requires a signed Data Use Agreement; for requests outside the EU, a separate DUA may be required. Commercial use is not unrestricted and would be assessed as part of the access procedure. Analysis code and non-identifying meta-data will be made publicly available where these do not contain individual-level participant information or create a risk of re-identification. The following dataset was generated: GeurtsDEM den OudenHEM SwartJC CookJL FallonSJ CoolsR FroböseM 2026Methylphenidate enhances or impairs the cognitive control of Pavlovian bias depending on working memory capacityRadboud University10.34973/8m6j-9695PMC1327174142299847

## Additional files

MDAR checklist

## Appendix 1

### Method 1: Overview of exclusion criteria

- (History of) psychiatric treatment.
- (History of) neurological treatment.
- (History of) endocrine treatment.
- (History of) autonomic failure (e.g. vasovagal reflex syncope).
- (History of) clinically significant hepatic, cardiac, obstructive respiratory, renal, cerebrovascular, metabolic or pulmonary disease.
- Family history of sudden death or ventricular arrhythmia.
- (History of) epilepsy.
- (History of) drug dependence (opiate, LSD, (meth)amphetamine, cocaine, solvents, or barbiturate), or alcohol dependence.
- Suicidality.
- Abnormal hearing or (uncorrected) vision.
- Use of MAO inhibitor, anaesthetic, anti-depressant, or antipsychotic drugs within the week prior to the start of the study.
- Use of psychotropic medication or of recreational drugs over a period of 24 hr prior to each test session, and use of alcohol within the last 24 hr before each measurement.
- Regular use of corticosteroids.
- Uncontrolled hypertension, defined as diastolic blood pressure at rest >95 mmHg or systolic blood pressure at rest >180 mmHg.
- Hypotension, defined as diastolic blood pressure <50 mmHg or systolic blood pressure <95 mmHg, or resting pulse rate <45 beats/min.
- Diabetes.
- Family history of schizophrenia, bipolar disorder, or major depressive disorder.
- Irregular sleep/wake rhythm (e.g. regular nightshifts or cross timeline travel).
- Possible pregnancy or breastfeeding.
- Lactose intolerance (placebo pill is a lactose product).

### Method 2: WM and impulsivity assessment

#### Listening span test

The listening span test was administered at the beginning of the second test session to obtain an estimate of participants’ WM capacity, as a putative proxy of baseline dopamine synthesis capacity (but see van den Bosch et al., 2023). During this test, participants listened to pre-recorded sentences and were given two tasks: They answered simple written multiple-choice questions about the content while remembering the last word of each sentence for later recall. The number of sentences on each trial (i.e. the span) increased up to 7 over the course of the task. Three series of the same span were conducted. The trial was coded as successful if the answers to the multiple-choice questions were correct and if all last words were remembered and reported in the correct order. Based on participants’ performance, a listening span was calculated ranging from 0 to a maximum of 7. The highest level for which two out of the three series were correctly remembered comprised the *basic span*. Half a point was added if one series of the following span was correctly completed, resulting in the measure of *total span*. For the listening span task, internal consistency has been shown to be adequate (0.70–0.90) based on coefficient alphas and split-half correlations (Salthouse and Babcock, 1991; Conway et al., 2005). Also test-retest correlations were high, approaching 0.70–0.80 across different studies varying in delay (Conway et al., 2005). Total listening span and total number of words recalled have both been shown to correlate positively with dopamine synthesis capacity (Cools et al., 2008; Landau et al., 2009). However, a large (n=100) recent study from our group did not show a link between WM capacity and dopamine synthesis capacity in the striatum, as measured with [18F]-FDOPA PET imaging.

Nevertheless, it is possible that the association with WM would hold when synthesis capacity was measured with [18F]-FMT PET imaging, which might have higher SNR and is sensitive to a different component of the metabolic pathway. Whether or not related to dopamine synthesis, listening span scores have repeatedly been shown to predict dopaminergic drug effects, justifying its inclusion as a relevant measure of between-subject variability (Kimberg and D’Esposito, 2003; Cools and D’Esposito, 2011; van der Schaaf et al., 2014).

#### Trait impulsivity

A series of questionnaires was completed by participants at home between the two test sessions. The trait impulsivity questionnaire was key to our research question and will be described in more detail below. The other questionnaire data were acquired for exploratory purposes, not pursued here, and are presented in Table 1. Trait impulsivity was assessed with the Barratt Impulsiveness Scale (BIS-11) (Patton et al., 1995). The BIS-11 is a self-report questionnaire, consisting of 30 statements tapping in common (non)impulsive behaviours and preferences, that participants rate on a 4-point Likert scale (‘never’ to ‘almost always’). Examples are ‘I buy things on impulse’ or ‘I am future oriented’. The BIS-11 total impulsivity scores reflect the tendency towards impulsivity. BIS-11 total scores have been shown in a large sample (N>1000) to have good internal consistency following a Cohen’s alpha of 0.83 and strong test-retest reliability at 1 month, evidenced by a correlation of 0.83 (Stanford et al., 2009). Scores have been found to be associated with dopamine D2/D3 receptor availability in the midbrain and enhanced dopamine release in the striatum (Lee et al., 2009; Buckholtz et al., 2010; Reeves et al., 2012; Kim et al., 2014) and has been shown to predict effects of MPH on learning (Clatworthy et al., 2009). This measure serves as a second putative proxy of baseline dopamine function for predicting effects of MPH. The effect sizes for the correlations between Barratt total scores and D2/D3 receptor availability ranged from small (Lee et al., 2009) to large (Buckholtz et al., 2010). Subjects completed the questionnaire at home between test days. Three participants did not complete one (n=2) or two (n=1) items of the questionnaire. For these items, we took the mean answer across all participants, allowing us to still include this data.

### Result 1: PIT effects are driven by valence but not magnitude of CS cues

We performed a model comparison (by means of the *anova* function in RStudio) to assess whether the data was better captured by a three level (averaging over the two appetitive and two aversive Valence Contexts, respectively) compared with a five level of Valence Context model. This was done based on previous studies that did not evidence significant differences of PIT effects for high vs. low appetitive Pavlovian cues, nor for high vs. low aversive Pavlovian (Huys et al., 2011; Geurts et al., 2013). We validated this for the current dataset where we compared the overall logistic mixed-level model described in the main paper model, where we collapse across high/low appetitive and high/low aversive cues, resulting in three valence levels (appetitive (1), neutral (0), aversive (–1)), with the same model with five valence levels (i.e. 2, 1, 0, –1, –2, respectively). Indeed, also in this dataset, the model with three-level Valence Context showed better model evidence than the five-level Valence Context model (AIC (delta AIC = –206), BIC (delta BIC = –207), and loglikelihood (delta loglikelihood = 103) were smaller for three-level model). For completeness, we also present the main figures on the PIT stage below for the five-level valence (Supplemental to Figure 1).

### Result 2: Control analyses

#### Instrumental training

Subjects learned to make correct instrumental actions during the instrumental training stage (Figure 1B, Main effect of trial number on accuracy: X^2^=37.2, p<1*10^–9^).

People had an initial bias to make a ‘Go’ response for approach trials (Figure 1B). This is reflected by the observation that on average, subjects were more accurate for Go compared to NoGo stimuli in the approach context, with no difference in the withdrawal context (Action Context × Stimulus type (Go vs. NoGo): X^2^=10.3, p=0.001; approach (Go/NoGo): X^2^=11.1, p=0.0009; withdrawal (Go vs. NoGo): X^2^=0.8, p=0.38). In line with this finding, subjects improved most for NoGo stimuli in the approach context (Action Context × Stimulus type (Go/NoGo) × Trial number X^2^=19.5, p=1*10^–5^).

To assess whether performance was similar across conditions at the end of learning, we assessed the final five trials for each instrumental cue. Indeed, there was no significant difference in plateau performance at the end of the instrumental training (Action Context × Stimulus type: X^2^=0.8, p=0.37; Action Context: X^2^=0.3, p=0.59; Stimulus type: X^2^=2.0, p=0.15; Figure 1A).

To account for any remaining potential differences in the degree of instrumental conditioning for different instrumental stimuli and subjects, we added the proportion of Go-choices for each stimulus for the final five trials of the instrumental stage (P(Go)Inst) as a covariate to the main PIT analysis. P(Go)Inst predicted choice in the PIT stage, evidencing that people maintained instrumental behaviour to the PIT stage (X^2^=2017, p<1*10^–16^). Importantly, addition of this covariate did not affect the main task effect (Action Context × Valence Context: X^2^=12.3, p<0.001) nor the main interaction of interest (Action Context × Valence Context × Drug × Listening Span: X^2^=13.2, p<0.001).

#### Pavlovian conditioning

Subjects performed highly accurately on the query trials during the Pavlovian stage, i.e., they successfully learned to select the stimuli associated with a higher number of points (median p(correct) after 25 presentations = 1, IQR: 0.96–1, range: 0.77–1, Figure 1C). Subjects further showed increased VAS liking scores from pre to post conditioning for the appetitive and decreased liking for the aversive Pavlovian CS (Time × Valence Context: X^2^=144.3, p<2*10^–16^; Main effect of Time: for appetitive Valence Context: mean difference: 0.2, X^2^=86.0, p<2*10^–16^; for the aversive Valence Context: mean difference: –0.2, X^2^=76.7, p<2*10^–16^; for the neutral Valence Context: mean difference = 0.03, X^2^=3.7, p=0.054).

#### Confound variables

Adding confound variables Drug Order, Day, Age, Gender, and Verbal intelligence to the main PIT analysis did not improve model evidence (all AIC and BIC of models with confounders >without confounders: all delta AIC < –27, all BIC differences < –208), nor did they interact with or change the main findings (Action Context × Valence Context × Drug × WM span: all X^2^>6.6, and all p<0.010). Moreover, adding Drug Order to the analyses of instrumental and Pavlovian stage did not improve model fit either (model with Drug Order >without: delta delta AIC = –23; BIC = –230).

##### Impulsivity

Inclusion of impulsivity as a covariate was planned a priori and consistently implemented in the other reports resulting from the same overarching study (Froböse et al., 2018; Cook et al., 2019; Rostami Kandroodi et al., 2021), particularly the study for which the current report is an eLife Research Advance (Swart et al., 2017). Indeed, both WM span and impulsivity were preregistered as potential factors (under secondary measures) that could mediate the effects of catecholamines on behaviour (see https://onderzoekmetmensen.nl/nl/trial/26989). The inclusion of WM span was based on evidence from PET imaging studies, demonstrating a link with dopamine synthesis capacity (Cools et al., 2008; Landau et al., 2009), whereas the inclusion of trait impulsivity was based on evidence from other PET imaging studies showing a link with dopamine (auto)receptor availability (Buckholtz et al., 2010; Kim et al., 2014; Lee et al., 2009; Reeves et al., 2012). The likelihood ratio test showed that adding Barratt did not improve the model (X^2^=6.5, p=0.59). For consistency with previous papers, we report the planned analyses, and additionally confirmed that the main finding is robust to exclusion of impulsivity scores, as the main four-way interaction remained significant (Action Context × Valence × Drug × WM span: X^2^=9.6, p=0.002). In addition, impulsivity and WM were uncorrelated (r_98_=−0.16, p=0.9), which rules out multicollinearity issues.

#### Deterministic PIT responding

Twenty-two subjects responded deterministically to the Valence Context for at least one of the Pavlovian cues. Our findings were robust to the exclusion of these subjects: Action-specific PIT effect (Action Context × Valence Context: X^2^=11.4, p=0.0008); Listening span-dependent effect of MPH on action-specific PIT (Action Context × Valence Context × Drug × Listening Span: X^2^=11.5, p=0.0007).

### Result 3: Mood and medical symptom rating

Mood ratings, heart rate, and blood pressure were monitored for safety reasons three times during each test day: (i) before capsule intake, (ii) upon start task battery, and (iii) upon completion of the task battery. The mood ratings consisted of the Positive and Negative Affect Scale (Watson et al., 1988) and the Bond and Lader Visual Analogues Scales (calmness, contentedness, alertness; Bond and Lader, 1974), as well as a medical Visual Analogues Scale (see below).

We assessed whether MPH affected mood and medical symptoms. For this control analysis, we performed a repeated-measures MANOVA using Pillai’s trace with the within-subject factors Time (baseline/start of testing/end of testing) and Drug (MPH/PLA), and dependent variables Positive Affect, Negative Affect, Calmness, Contentedness, Alertness, and Medical Symptoms. To assess whether our covariates of interest (WM span, impulsivity) were correlated to the effects of MPH on mood/medical symptoms, these covariates were added to this initial MANOVA. Significant effects were further explored with Bonferroni corrected repeated-measures ANOVA comparing pre (T1) vs. post (T2, T3) intake (Helmert contrast), where alpha = 0.05/6 ≈0.008. Finally, we computed a MANOVA of baseline (T1) effects only with factor Drug and the same dependent variables, to verify absence of significant baseline differences in these ratings. Greenhouse-Geisser correction was applied when the assumption of sphericity was not met.

MPH affected these self-report ratings (Time × Drug: V=0.39, F(12,87) = 4.7, p<0.001), in the absence of baseline (T1) differences between the MPH and placebo groups (Drug: V=0.05, F(6,93) = 0.8, p=0.6). The effects of MPH on the self-report ratings did not change when Listening Span and Impulsivity (BIS) were included as covariates, nor did either of these covariates significantly interact with the Time × Drug interaction (Listening Span × Time × Drug: V=0.10, F(12,85) = 0.8, p=.6; Impulsivity × Time × Drug: V=0.19, F(12,85) = 1.6, p=0.10), which suggests that the MPH-induced changes in mood and medical symptoms were orthogonal to the Listening Span-dependent MPH effects we observed in the task. Relative to placebo, after capsule intake MPH increased Positive Affect (F(1,98) = 22.3, p<0.001), Alertness (F(1,98) = 17.3, p<0.001), and Medical Symptoms (F(1,98) = 8.3, p=0.005), decreased Calmness (F(1,98) = 8.0, p=0.006), and did not significantly affect Contentedness (F(1,98) = 1.5, p=0.23) and Negative Affect (F(1,98) = 0.3, p=0.6). Note that these effects were also reported in the other papers based on this study, though for a slightly different subset of participants, as we excluded three further participants here for technical problems with the PIT task data acquisition.

**Table:**

Medical visual analogue scale	
No headache	Strong headache
No muscle pain	Strong muscle pain
No dry mouth	Very dry mouth
Not dizzy	Very dizzy
No abdominal pain	Strong abdominal pain
No joint pain	Strong joint pain
No trouble breathing	Trouble breathing
No throat pain	Strong throat pain
No chest pain	Strong chest pain
No eye problems	Strong eye problems
