# Supplementary material for: Methylphenidate enhances or impairs the cognitive control of Pavlovian bias depending on working memory capacity
Source: eLife. 2026 Jun 16;13:RP98917. doi: 10.7554/eLife.98917 (PMC13271741; doi:10.7554/eLife.98917)
Supplement: Peer review history

# Editor report: eLife Assessment

**DOI:** 10.7554/eLife.98917.3.sa0

**Authors:** Mitul Mehta

**Affiliations:** King's College London United Kingdom

**Keywords:** Compelling, Important

**Important** findings from this study include clear evidence of the impact of methylphenidate on cognitive control over Pavlovian biasing of actions and decision-making in humans, in a manner dependent on baseline working memory capacity. The design used drug dosing after learning, allowing a **compelling** test of the influence of catecholamines on decision processes independent of learning. The task is very well designed, using a combination of aversive and appetitive Pavlovian to Instrumental Transfer, and the in-depth behavioural analysis extends the authors' previous work, which will be of interest to those working in cognitive psychopharmacology. The results challenge the view that catecholamines operate by modulating behavioural invigoration alone.

# Referee report: Reviewer #1 (Public review)

**DOI:** 10.7554/eLife.98917.3.sa1

Summary:

The authors use methylphenidate (MPH) administration after learning a Pavlovian-to-instrumental transfer (PIT) task to parse decision making from instrumental influences. While the main pharmacological effects were null, individual differences in working memory ability moderated the tendency of MPH to boost cognitive control in order to override PIT-biased instrumental learning. Importantly, this working memory moderator had symmetrical effects in appetite and aversive conditions, and these patterns replicated within each valence condition across different values of gain/loss (Fig S1c), suggesting a reliable effect that is generalized across instances of Pavlovian influence.

Strengths:

The idea of using pharmacological challenge after learning but prior to transfer is a novel technique that highlights the influence of catecholamines on the expression of learning under Pavlovian bias, and importantly it dissociates this decision feature from the learning of stimulus-outcome or action-outcome pairings.

Comments on revisions:

I have no further recommendations or concerns.

# Referee report: Reviewer #2 (Public review)

**DOI:** 10.7554/eLife.98917.3.sa2

Summary:

In this study, Geurts et al. investigated the effects of the catecholamine reuptake inhibitor methylphenidate (MPH) on value-based decision making using a combination of aversive and appetitive Pavlovian to Instrumental Transfer (PIT) in a human cohort. Using an elegant behavioural design they showed a valence- and action-specific effects of Pavlovian cues on instrumental responses. Initial analyses showed no effect of MPH on these processes. However the authors performed a more in-depth analysis and demonstrated that MPH actually modulates PIT in action-specific manner, depending on individual working memory capacities. The authors interpret that as an effect on cognitive control of Pavlovian biasing of actions and decision-making more than an invigoration of motivational biases.

Strengths:

A major strength a this study is its experimental design. The elegant combination of appetitive and aversive Pavlovian learning with approach/avoidance instrumental actions allows the authors to precisely investigate the differential modulation of value-based decision making, depending on the context and environmental stimuli. Importantly, MPH was only administered after Pavlovian and instrumental learning, restricting the effect to PIT performance only. Finally, the use of a placebo-controlled crossover design allows within-comparisons between the PIT effect under placebo and MPH and the investigation of the relationships between working memory abilities, PIT and MPH effects.

Weaknesses:

Previous weaknesses regarding the neurobiological circuits underlying such effects and the possible role of dopamine vs noradrenaline have been clearly discussed in the new version of the manuscript.

Comments on revisions:

The authors answered my previous points. The changes to the manuscript clearly improve the clarity of the results and the strength of the study.

# Author comment: Author response

**DOI:** 10.7554/eLife.98917.3.sa3

**Authors:** Dirk EM Geurts, Hanneke EM den Ouden, Jennifer C Swart, Monja I Froböse, Sean Fallon, Jennifer L Cook, Roshan Cools

**Affiliations:** Radboud University Medical Center Nijmegen Netherlands; Radboud University Nijmegen Nijmegen Netherlands; Radboud Universiteit Nijmegen Donders Institute for Brain Cognition and Behaviour Nijmegen Netherlands; Radboud University Nijmegen Nijmegen Netherlands; School of Psychology, University of Plymouth Plymouth United Kingdom; University of Birmingham Birmingham United Kingdom; Donders Institute for Brain, Cognition and Behaviour, Radboud University Nijmegen Nijmegen Netherlands

The following is the authors’ response to the original reviews.

**Public Reviews:**

**Reviewer #1 (Public review):**

Summary:

The authors use methylphenidate (MPH) administration after learning a Pavlovian to instrumental transfer (PIT) task to parse decision making from instrumental influences. While the main effects were null, individual differences in working memory ability moderated the tendency of MPH to boost cognitive control in order to override PIT-biased instrumental learning. Importantly, this working memory moderator had symmetrical effects in appetite and aversive conditions, and these patterns replicated within each valence condition across different values of gain/loss (Fig S1c), suggesting a reliable effect that is generalized across instances of Pavlovian influence.

Strengths:

The idea of using pharmacological challenge after learning but prior to transfer is a novel technique that highlights the influence of catecholamines on the expression of learning under Pavlovian bias, and importantly it dissociated this decision feature from the learning of stimulus-outcome or action-outcome pairings.

We thank the reviewer for highlighting the timing of the pharmacological intervention as a strength for this study and for the suggested improvements for clarification.

Weaknesses:

While the report is largely straightforward and clearly written, some aspects may be edited to improve the clarity for other readers.

(1) Theoretical clarity. The authors seem to hedge their bets when it comes to placing these findings within a broader theoretical framework.

Our findings ask for a revision of theories on how catecholamines are involved in instantiation of Pavlovian biases in decision making. The reviewer rightly notices that we offer three routes to modify current theory to be able to incorporate our findings. Briefly, these routes discuss catecholaminergic modulation of Pavlovian biases (i) through modulation of the putative striatal ‘origin’ of Pavlovian biases, (ii) through top-down control, primarily relying on prefrontal processes, and (ii) a combination of the two, where catecholamines regulate the balance between these striatal and frontal processes.

Given the systemic nature of the pharmacological manipulation, we cannot dissociate between these three accounts. We believe that discussing these possible explanations enriches our Discussion and strengthens our recommendation in the ultimate paragraph to use pharmacological neuroimaging studies to arbitrate between these options. In the revision, we have made this line of reasoning more clear, in part by adding guiding titles to the Discussion section and adding a summary paragraph in the Discussion (Discussion, page 9-12).

(2) Analytic clarity: what's c^2?

C^2 seems a technical pdf conversion error problem: all chi-squares (Χ2) have been converted to C2. This is now corrected in our revision.

**Reviewer #2 (Public review):**

Summary:

In this study, Geurts et al. investigated the effects of the catecholamine reuptake inhibitor methylphenidate (MPH) on value-based decision making using a combination of aversive and appetitive Pavlovian to Instrumental Transfer (PIT) in a human cohort. Using an elegant behavioural design they showed a valence- and action-specific effects of Pavlovian cues on instrumental responses. Initial analyses show no effect of MPH on these processes. However the authors performed a more in-depth analysis and demonstrated that MPH actually modulates PIT in actionspecific manner depending of individual working memory capacities. The authors interpret that as an effect on cognitive control of Pavlovian biasing of actions and decision making more than an invigoration of motivational biases.

Strengths:

A major strength of this study is its experimental design. The elegant combination of appetitive and aversive Pavlovian learning with approach/avoidance instrumental actions allows to precisely investigate the different modulation of value-based decision making depending on the context and environmental stimuli. Important MPH is only administered after Pavlovian and instrumental learning, restricting the effect on PIT performance only. Finally, the use of a placeboontrolled crossover design allows within-comparisons between PIT effect under placebo and MPH and the investigation of the relationships between working memory abilities, PIT and MPH effects.

We thank the reviewer for highlighting the experimental design as a strength for this study and the suggested improvements for clarification.

Weaknesses:

As authors stated in their discussion, this study is purely correlational and their conclusions could be strengthened by the addition of interesting (but time- and resource-consuming) neuroimaging work.

We employ a pharmacological intervention within a randomized placebo controlled cross-over design, which allows for causal inferences with respect to the placebo-controlled intervention. Thus, the reported interactions of interest include correlations, but these are causally dependent on our intervention.

Perhaps the reviewer refers to the implications of our findings for hypotheses regarding neural implementation of Pavlovian bias-generation. Indeed, based on our data we are not able to arbitrate between frontal and striatal accounts, due to the systemic nature of the pharmacological intervention. Thus, we agree with the reviewer that neuroimaging (in combination with for example brain stimulation) would be a valuable next step to identify the neural correlates to these pharmacological intervention effects, to dissociate between frontal and striatal basis of the effects. In the revision, as per our reply to reviewer 1, we have made this line of reasoning more clear, in part by adding guiding titles to the Discussion section and adding a summary paragraph in the Discussion (Discussion, page 9-12).

The originality of this work compared to their previous published work using the same cohort could also be clarified at different stages of the article, as I initially wondered what was really novel. This point is much clearer in the discussion section.

As recommended, we brought forward parts of the Discussion that clarify the originality of the current experiment to the introduction (page 4/5) and result section (page 8).

A point which, in my opinion, really requires clarification is when the working memory performance presented in Figure 2B has been determined. Was it under placebo (as I would guess) or under MPH? If it is the former, it would be also interesting to look at how MPH modulates working memory based on initial abilities.

We now clarified that working memory span was assessed for all participants on Day 2 prior to the start of instrumental training (as illustrated in figure 1A). Importantly, this was done prior to ingestion of the drug or placebo (which subjects received after Pavlovian training, which followed the instrumental training). This design also precludes an assessment of the effects of MPH on working memory capacity.

A final point is that it could be interesting to also discuss these results, not only regarding dopamine signalling, but also including potential effect of MPH on noradrenaline in frontal regions, considering the known role of this system in modulating behavioural flexibility.

We indeed focus our Discussion more on dopamine than on noradrenaline. Our revision now also discusses noradrenaline in light of our frontal control hypothesis and the recommendation, in future studies, to use a multi-drug design, incorporating, for example, a session with the drug atomoxetine, which modulates cortical catecholamines, but not striatal dopamine (Discussion, page 12).

**Reviewer #3 (Public review):**

The manuscript by Geurts and colleagues studies the effects of methylphenidate on Pavlovian to instrumental transfer in humans and demonstrates that the effects of the drug depend on the baseline working memory capacity of the participants. The experiment used a well established cognitive task that allows to measure the effects of Pavlovian cues predicting monetary wins and losses on instrumental responding in two different contexts, namely approach and withdraw. By administering the drug after participants went through the instrumental and Pavlovian learning phases of the experiment, the authors limited the effects of the drug to the transfer phase in extinction. This allowed the authors to make inference about the invigorating effects of the cues independently from any learning bias. Moreover, the authors employed a within subject design to study the effect of the drug on 100 participants, which also allows to detect continuous between-subject relationships with covariates such as working memory capacity.

The study replicates previous findings using this task, namely that appetitive cues promote active responding, and aversive cues promote passive responding in an approach instrumental context, whereas the effect of the cues reverses in a withdraw instrumental context. The results of the methylphenidate manipulation show that the drug decreases the effects of the Pavlovian cues on instrumental responding in participants with low working memory capacity but increases the Pavlovian effects in participants with high working memory capacity. Importantly, in the latter group, methylphenidate increases the invigorating effect of appetitive Pavlovian cues on active approach and aversive Pavlovian cues on active withdrawal as well as the inhibitory effects of aversive Pavlovian cues on active approach and appetitive Pavlovian cues on active withdrawal. These results cannot be explained if catecholamines are just involved in Pavlovian biases by modulating behavioral invigoration driven by the anticipation of reward and punishment in the striatum, as this account can't account for the reversal of the effects of a valence cue on vigor depending on the instrumental context.

In general, I find the methods of this study very robust and the results very convincing and important. However, I have some concerns:

We thank the Reviewer for highlighting the robustness of the methods and the importance of the results. We are glad to shortly address the concerns here and have incorporated these in our revision.

I am not convinced that the inclusion of impulsivity scores in the logistic mixed model to analyze the effects of methylphenidate on PIT is warranted. The authors do not show whether inclusion of this covariate is justified in terms of BIC. Moreover, they include this covariate but do not report the effects. Finally, it is possible that impulsivity is correlated with working memory capacity. In that case, multicollinearity may impact the estimation of the coefficient estimates and may inflate the p-values for the correlated covariates. Are the reported results robust when this factor is not included?

With regard to the inclusion of impulsivity we first like to mention that this inclusion in our analyses was planned a priori and therefore consistently implemented in the other reports resulting from the overarching study (Froböse et al., 2018; Cook et al., 2019; Rostami Kandroodi et al., 2021), especially the study with regard to which the current report is an e-life research advance (Swart et al., 2017). Moreover, we preregistered both working memory span and impulsivity as potential factors (under secondary measures) that could mediate the effects of catecholamines (see https://onderzoekmetmensen.nl/nl/trial/26989). The inclusion of working memory span was based on evidence from PET imaging studies demonstrating a link with dopamine synthesis capacity (Cools et al., 2008; Landau et al, 2009), whereas the inclusion of trait impulsivity was based on evidence from other PET imaging studies showing a link with dopamine (auto)receptor availability (Buckholtz et al., 2010; Kim et al., 2014; Lee et al., 2009; Reeves et al., 2012). Although there was no significant improvement for the model with impulsivity compared with the model without impulsivity, we feel that we should follow our a priori established analyses.

We can confirm that impulsivity and working memory were not correlated in this sample (r98=-0.16, p=0.88), which rules out multicollinearity.

Most importantly, results are robust to excluding impulsivity scores as evidenced by a significant four-way interaction from the omnibus GLMM without impulsivity (Action Context x Valence x Drug x WM span: X^2^ = 9.5, p=0.002). We will report these findings in the revised manuscript. We now added the text to the Supplemental Results: Control analyses, page 28.

The authors state that working memory capacity is an established proxy for dopamine synthesis capacity and cite some studies supporting this view. However, the authors omit a recent reference by van den Bosch et al that provides evidence for the absence of links between striatal dopamine synthesis capacity and working memory capacity. The lack of a robust link between working memory capacity and dopamine synthesis capacity in the striatum strengthens the alternative explanations of the results suggested in the discussion.

We agree with the Reviewer that the lack of a robust link between working memory capacity and dopamine synthesis capacity in the striatum, as measured with [^18^F]-FDOPA PET imaging, is lending support for the proposed hypothesis incorporating a broader perspective on Pavlovian bias generation than the dopaminergic direct/indirect pathway account (although it is possible that the association will hold in a larger sample when synthesis capacity is measured with [^18^F]-FMT PET imaging, which is sensitive to a different component of the metabolic pathway). We will indeed incorporate in our planned revision the findings from our group reported in van den Bosch et al (2022).

See Supplemental methods 2: Working memory and impulsivity assessment, page 26.

** Recommendations for the authors:**

**Reviewer #1 (Recommendations for the authors):**

(1) Theoretical clarity. Some aspects of the paper are ideally clear: Figure 1 clearly explains the paradigm. The general take-home message is clearly described in the last line of the abstract, the last line of the introduction, the first line of the discussion, and throughout other places in the discussion. Yet the authors seem to hedge their bets when it comes to placing these findings within a broader theoretical framework.

The discussion includes many possible theoretical interpretations of the findings, which is laudable, but many readers may get lost in this multitude (particularly anyone who isn't an RL/DA aficionado). The group's prior work (i.e. striatal hypothesis) is first described, followed by a rather complex breakdown of valenceaction tendencies, then the seemingly preferred explanation for the current study (i.e. cognitive control hypothesis) is advanced as "an alternative account ...". This is followed by a third, more complex idea (i.e. cortico-striatal balance hypothesis), then the paper ends. A reader may be forgiven for skimming through this discussion and not having a clear idea of how to frame these effects. I think some subheaders would help, as well as clearer labeling of the theoretical interpretations in line with a more authoritative description of the author's preferred interpretation of the empirical effects.

Our findings ask for a revision of theories on how catecholamines are involved in instantiation of Pavlovian biases in decision making. The reviewer rightly notices that we offer three routes to modify current theory to be able to incorporate our findings. Briefly, these routes discuss catecholaminergic modulation of Pavlovian biases (i) through modulation of the putative striatal ‘origin’ of Pavlovian biases, (ii) through top-down control, primarily relying on prefrontal processes, and (ii) a combination of the two, where catecholamines regulate the balance between these striatal and frontal processes.

Given the systemic nature of the pharmacological manipulation, we cannot dissociate between these three accounts. We believe that discussing these possible explanations enriches our Discussion and strengthens our recommendation in the ultimate paragraph to use pharmacological neuroimaging studies to arbitrate between these options. In the revision, we have made this line of reasoning more clear, in part by adding guiding titles to the Discussion section and adding a summary paragraph in the Discussion (Discussion, page 9-12).

(2) All statistical effects are presented as c^2 with no df. The methods only describe LMER and make no mention of what the c^2 measure represents.

C^2 seems a technical pdf conversion error problem: all chi-squares (Χ2) have been converted to C2. This is now corrected in our revision.

**Reviewer #2 (Recommendations for the authors):**

Few minor points:

Figure 2A is not cited in the text I think

Checked and changed.

Figure 2C: "C" is not present in the figure. Also I could not see the data corresponding at MPH-Approach context in Neutral Pavlovian condition but I think it is probably masked by another curve.

Checked and changed. Indeed, the one curve is masked by the other curve.

As I stated in the public review, a clarification or more detailed analysis of working memory performance depending on if it was measured under MPH or placebo could be a plus.

Changed this (see public review reply).

I did not see any statement about the availability of data but I may have missed it.

Yes, the statement can be found:

Methods, page 13: Data and code for the study are freely available at https://data.ru.nl/collections/di/dccn/DSC_3017031.02_734.

**Reviewer #3 (Recommendations for the authors):**

The authors should check that inclusion of impulsivity in the logistic mixed model is justified and if it is justified make sure that multicollinearity is not problematic.

See answer to public review for convenience reiterated below:

With regard to the inclusion of impulsivity we first like to mention that this inclusion in our analyses was planned a priori and therefore consistently implemented in the other reports resulting from the overarching study (Froböse et al., 2018; Cook et al., 2019; Rostami Kandroodi et al., 2021), especially the study with regard to which the current report is an e-life research advance (Swart et al., 2017). Moreover, we preregistered both working memory span and impulsivity as potential factors (under secondary measures) that could mediate the effects of catecholamines (see https://onderzoekmetmensen.nl/nl/trial/26989). The inclusion of working memory span was based on evidence from PET imaging studies demonstrating a link with dopamine synthesis capacity (Cools et al., 2008; Landau et al, 2009), whereas the inclusion of trait impulsivity was based on evidence from other PET imaging studies showing a link with dopamine (auto)receptor availability (Buckholtz et al., 2010; Kim et al., 2014; Lee et al., 2009; Reeves et al., 2012). Although there was no significant improvement for the model with impulsivity compared with the model without impulsivity, we feel that we should follow our a priori established analyses.

We can confirm that impulsivity and working memory were not correlated in this sample (r98=-0.16, p=0.88), which rules out multicollinearity.

Most importantly, results are robust to excluding impulsivity scores as evidenced by a significant four-way interaction from the omnibus GLMM without impulsivity (Action Context x Valence x Drug x WM span: X^2^ = 9.5, p=0.002). We will report these findings in the revised manuscript. We now added the text to the Supplemental Results Control analyses, page 28.

I would recommend that the authors make clear that the effects of methylphenidate are dependent on working memory capacity in the first sentence of the fore last paragraph of the introduction on page 4.

Changed this accordingly, see Introduction, page 5.

I would make sure that the text in the figures is readable without needing to enlarge the figures. I would also highlight the significant effects in the figures.

We changed the font size accordingly and added significance statements to the caption, because depicting the significance of a four-way interaction including one continuous variable is not straightforward.

The distributions of p(Go) by conditions such as in figure 1D or 2A are very intuitive. Figure 2B is very informative as it shows the continuous effects of working memory capacity on the PIT effect. I would add (in figure 2 or in the supplement) a plot of the p(Go) with a tertile split based on working memory. Considering that the correspondent analysis is being reported, having the plot would strengthen and simplify the understanding of the results.

The continuous effects of working memory are based on WM values on the listening span ranging from 2.5-7, in steps of 0.5, resulting in 10 different values. A tertile split would result in binning these into two bins of three values, and one bin of four values. Given that all of the datapoints for this tertile split are already presented in the current figures, we strongly prefer not to include this additional figure.

I would add some sentences in the results section (and maybe in the discussion if needed) addressing the results that the effect of Valence by drug by WM span is only significant in the withdrawal context but not in the approach context.

We now added an emphasis on the specifically significant drug effects in withdrawal in the Results section, page 8.
